# Surgical treatment of costal cartilage fractures with titanium plate internal fixation

**DOI:** 10.1186/s13019-022-01801-1

**Published:** 2022-03-28

**Authors:** Yang Li, Yonghong Zhao, Yi Yang, Weiming Wu, Xiang Guo, Tiancheng Zhao

**Affiliations:** Department of Thoracic Surgery, Shanghai Jiaotong University Affiliated Sixth People’s Hospital, Shanghai, 200233 China

**Keywords:** Chest trauma, Costal cartilage fracture, Internal fixation

## Abstract

**Background:**

This study aim to evaluate surgical procedures for titanium plate internal fixation of costal cartilage fractures with displacement or nonunion.

**Methods:**

From January 2019 to October 2020, 13 patients with costal cartilage fractures were treated with titanium plate internal fixation in the thoracic surgery department of the Shanghai Sixth People’s Hospital. Pain severity scale scores and respiratory function were evaluated preoperatively and postoperatively. All the patients had a 6-month follow-up for treatment evaluation.

**Results:**

The mean hospital length of stay was 10.7 days. A statistically significant difference (*P* < 0.05) was found between preoperative and postoperative pain severity scores (7.69 vs. 5.00). VC (24.6% vs. 44.5%) and FEV1 (25.3% vs. 44.0%) were also significantly different before operation and after operation (*P* < 0.05). At follow-up, healing of the nonunion or fracture was confirmed in all the cases.

**Conclusion:**

The rigid titanium plate application ensured a safe and easy management of costal cartilage fractures and nonunion with a good prognosis as compared with other methods.

## Background

Costal cartilage fracture accounts for about 7% of all admissions for rib fractures [1]. It is not uncommon and is often caused by direct, frontal, blunt trauma to the sternum. Costal cartilage fractures are common in high-energy blunt chest trauma and often occur with multiple consecutive rib fractures. We knew less about costal cartilage fractures. In our work, we found a few cases with instability or obvious displacement can lead to severe disabling conditions, including severe chest pain, dyspnea, persistent cough, and chest wall paradoxical motion. The diagnosis and treatment of costal cartilage fractures have rarely been discussed systematically in the literature. Some case-report articles described solitary costal cartilage fracture heal with conservative or surgical management [2, 3]. But these are still far from enough.

We evaluated the status of costal cartilage fractures by using computed tomography (CT) and three-dimensional (3-D) reconstruction imaging. Patients with costal cartilage displacement underwent surgery with a titanium plate, with screws rigidly fixed to the plate. Preoperative and postoperative respiratory function and pain scores were assessed, the purpose of this study was to evaluate the therapeutic effect of this internal fixation technique.

## Methods

From January 2019 to October 2020, 13 patients with costal cartilage fractures were treated with titanium plate internal fixation in our thoracic surgery department. This article has been approved by Ethics Committee of Shanghai Sixth People’s Hospital (No. 2019-138-(1)).The operative criterion was a disabling nonunion or obvious displaced fractures of costal cartilage. The exclusion criteria were inability to provide informed consent or health status that ruled out general anesthesia.

**Fig. 1:**
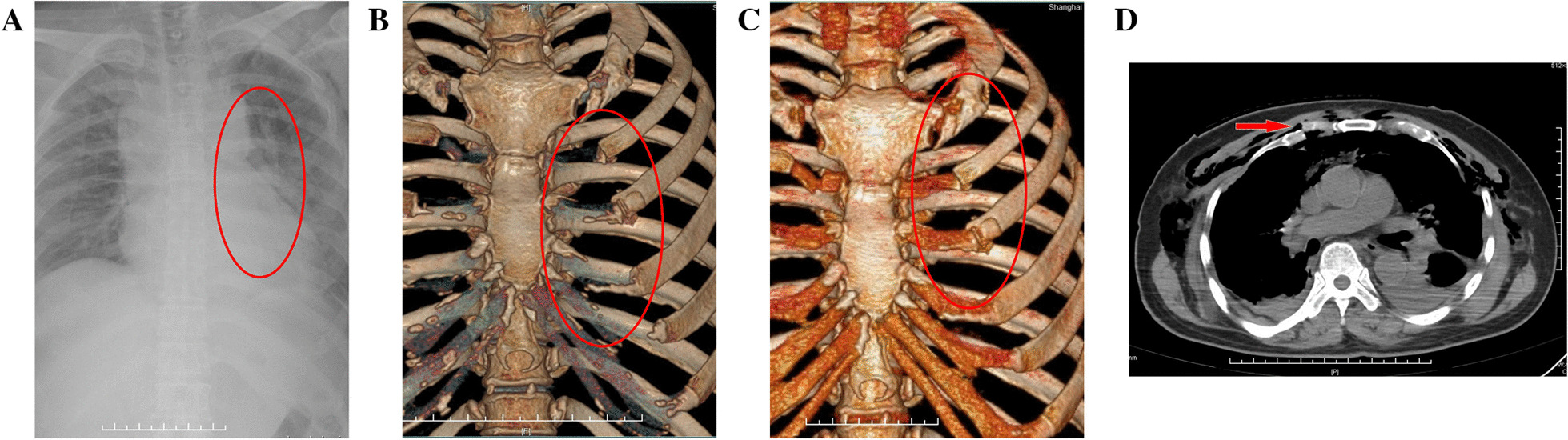
Showing an x-ray film and two kinds of CT images of costal cartilage fractures from the same patient. **A** Showing costal cartilage fractures are almost impossible to show on normal X-ray. **B** Showing costal cartilage fractures are occult on normal 3D CT image. **C**, **D** Showing costal cartilage fractures clearly on transverse section of CT image and cartilage specified 3D reconstruction CT image

**Fig. 2:**
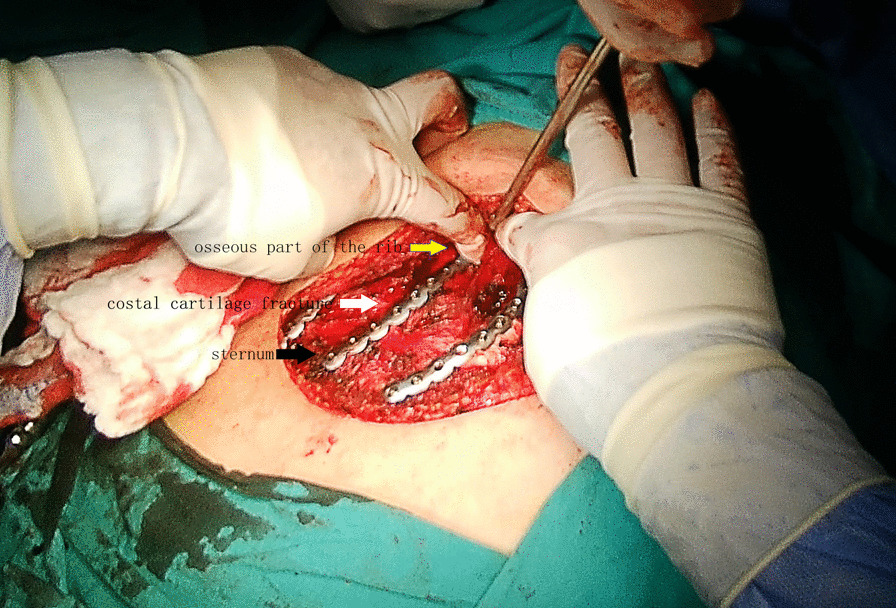
CT Showing a cured costal cartilage fracture 6 months after operation

All the patients underwent CT scan and 3-D reconstruction of the costal cartilage preoperatively. This is useful in evaluating the fracture pattern and locating the fracture position. Figure 1A–C shows three different images from the same patient. Figure 1D shows costal cartilage fractures clearly on transverse section of CT image. Pain severity scale scores were measured both preoperatively and postoperatively. The patients were asked to grade their pain on a scale of 0 to 10, with 0 being “none” and 10 being the worst pain they had ever felt. The pain index was evaluated before surgery and on the third day after surgery. General anesthesia and single-lumen endotracheal intubation were suitable for the operation. The supine position and slight overextension of the chest wall were useful in repositioning the fracture. The patients returned to their daily activities soon after surgery. All the patients had a 6-month follow-up for evaluation of recovery by using radiography or CT. Figure 2 shows a patient 6 months after internal fixation of costal cartilage fractures.

**Fig. 3:**
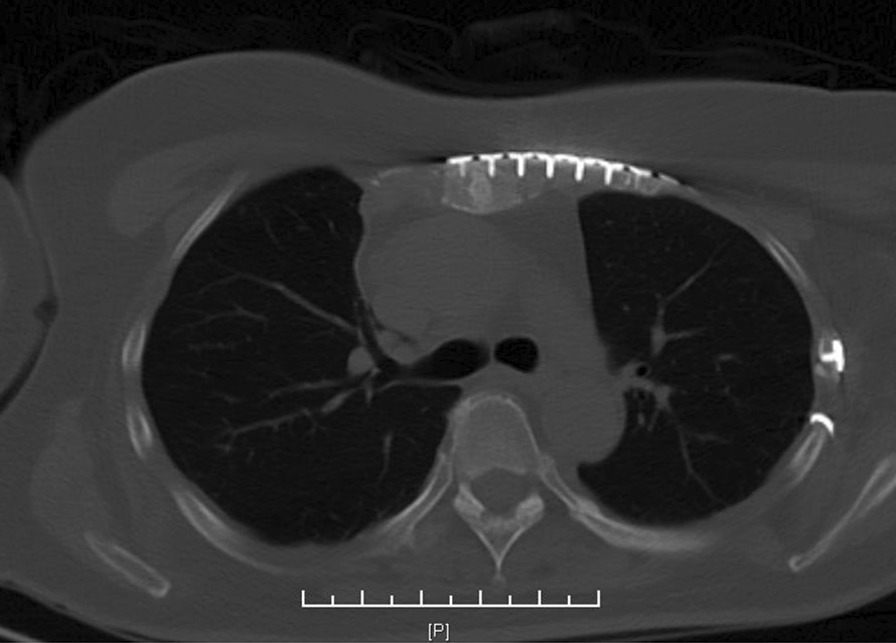
Intraoperative photo showing multiple left-sided costal cartilage fractures stabilized by long threaded plates. The pectoralis major muscle is retracted laterally. The plates are attached by screws medially to the sternum and laterally to the osseous part of the rib, with screws through the cartilage

The internal fixation device we used consisted of a titanium plate and screws. According to different fracture patterns, we bent a preforming linear plate to match the shape. The overall thickness of the plate was 2.4 mm in all the patients. Figure 3 is an intraoperative photo showing multiple left-sided costal cartilage fractures stabilized by long threaded plates. The pectoralis major muscle is retracted laterally. The plates are attached by screws medially to the sternum and laterally to the osseous part of the rib, with screws through the cartilage. The implant was produced by DePuy Synthes, USA.

The data were analyzed with SPSS version 19.0 (SPSS Inc., Chicago, IL, USA). The data with normal distribution were presented as mean ± standard deviation (SD) and independent sample t test, and enumeration data were expressed as a rate χ^2^ test. *P* values of < 0.05 were considered statistically significant.

## Results

General clinical characteristics of patients are shown in Table 1.Thirteen patients underwent the procedure, 9 of whom were male (69.2%). The mean age was 47 years (range 21–71 years; Table 1). The mean duration between trauma and surgery was 3.25 days (range 1–7 days). Traffic accidents were the most common injury mechanism (10 patients, 76.9%).

**Table 1:** General clinical characteristics of patients

	n = 13
Gender	
Male	9(69.2%)
Female	4(30.8%)
Average number of fractures	3.5
Age	47(min 29, max 63)
Operation time (min)	85.32 ± 15.45
Hospital stay (day)	10.72 ± 1.46

The mean operative time was 85.32 ± 15.45 min. No operation-related complications were observed. The mean hospital stay was 10.7 days. A statistically significant difference (*P* < 0.05) was found between preoperative and postoperative pain severity scores (7.69 ± 1.31 vs. 5.00 ± 1.22, respectively). At follow-up, healing of fracture was confirmed in all the cases. No removal of metalwork was performed in the follow-up period. All the patients were followed up after operation for 6 months and underwent reevaluation for lung function with chest radiography, which revealed healed bones, callus formation, no nonunion, and displacement. No failure of internal fixation or migration of the internal fixation device was observed. All the patients were evaluated for pulmonary function before operation and on postoperative day 7 (FVC: forced vital capacity, FEV1: forced expiratory volume in 1 s). The European Community of Coal and Steel (ECCS) formula was used to calculate the predictive value of the pulmonary function of the patients. We compared the measured and predicted values, and analyzed them. Studies have shown that the mean FVC values before operation and on postoperative day 7 accounted for 24.64% ± 3.60% and 44.58% ± 3.15% of the predicted values, respectively. FEV1 respectively accounted for 25.25% ± 3.51% and 44.04% ± 3.10% of the predicted values. Compared with pulmonary function before operation, that on postoperative day 7 significantly improved (*P* < 0.05; Table 2).

**Table 2:** Respiratory function and pain scale result

	Preoperative	Postoperative
FVC	24.64% ± 3.60%	44.58% ± 3.15%
FEV1	25.25% ± 3.51%	44.04% ± 3.10%
Pain index	7.69 ± 1.31	5.00 ± 1.22

## Discussion

The most common pathologies in thorax traumas are multiple rib fractures, but costal cartilage fractures could be easily overlooked. To the best of our knowledge, few series that aimed specifically to diagnose and manage COSTAL CARTILAGE fractures are available in the literature [3, 4].

It is difficult to detect costal cartilage fractures by ordinary X-rays in the diagnosis of costal cartilage fractures. Besides, it is also difficult to detect costal cartilage fractures by the bone window reconstruction in chest CT. Therefore, in case of violent trauma to the anterior chest wall, great importance should be attached to cross-sectional CT images to avoid missing costal cartilage fractures. In case of suspected costal cartilage fractures or if cross-sectional images suggest costal cartilage fractures, three-dimensional (3D) imaging of cartilage may be performed to show costal cartilage fractures. It is also reported in some literatures that ultrasonic diagnosis can be used to help detect costal cartilage fractures. Since bedside ultrasound can also be helpful in locating the fracture, it was also performed for the surgical localization of costal cartilage fractures before the operation [5].

Since the 1st–7th ribs and the sternum are connected by the costal cartilage, the 8th-10th costal cartilages form a costal arch, and the 11th and 12th ribs are floating ribs, the 8th-12th costal cartilages are believed to have little impacts on the stability of the chest wall. We mainly fixed the fractures of the 1st–7th costal cartilages [6]. We chose an arc incision from the sternum to the lower margin of the pectoralis major, which could fully expose the sternum end and the rib-costal cartilage junction after the pectoralis major was dissociated, so as to obtain a good surgical field of view. At present, no literature or manufacturer’s technical document explains whether the internal fixation with plate and screw can be performed directly on costal cartilages. The technical document of Johnson and Johnson just recommends attaching the internal fixator to the bony component. Therefore, we chose to fix both ends of internal fixation materials to the surface of ribs and the sternum, respectively [7]. Meanwhile, costal cartilages were fixed with screws after reduction. In this way, the separation of the fracture site of costal cartilages could be avoided to the greatest extent, thus lowering the probability of injuring the internal mammary artery and ensuring better blood supply to the fracture site. In fokin’s paper, the plate was fixed to the costal cartilage with strapping rather than screws. Nevertheless, he did not mention the reason for this operation [8]. During our follow-up visits, all costal cartilage fractures healed well without re-displacement or bone ununion.

It has been proved in our previous studies that the fractures of multiple continuous ribs may cause severe impairment of pulmonary function (PF) [9, 10]. Meanwhile, it has also been proved that the internal fixation of rib fracture can significantly improve the impaired PF in the acute phase, which has also been confirmed by a great number of studies [11–13]. In this study, we confirmed that costal cartilage fractures may also cause the impaired integrity of thoracic cage [14]. Since the costal cartilage is the starting point of the connection between the sternum and ribs, a fracture and dislocation of the costal cartilage may easily lead to impaired respiratory function. Even though only a small number of (2–3) costal cartilages are fractured, the local collapse of the thoracic cage may also be caused, which may also directly lead to a severe impairment of lung function. After fracture fixation, the FVC and FEV1 of all patients were significantly improved. There were significant differences in these indicators before and after the operation (*P* < 0.05). Meanwhile, since the fixation reduced the stimulation of the intercostal nerve by the movement of the fracture site of ribs, patients’ pains were significantly improved by internal fixation; and there was significant difference in the pain score before and after the operation (*P* < 0.05).

## Conclusion

The use of a titanium plate and locking screws offers a reliable method for the successful treatment of costal cartilage fractures. We believe that internal fixation is a proper surgical treatment technique for costal cartilage fractures and displacement.

## Data Availability

All data and materials in this article are available.

## Abbreviations

CT: Means computed tomography
FVC: Means forced vital capacity
FEV1: Means forced expiratory volume in 1 s
